# Neuromusculoskeletal Control for Simulated Precision Task versus Experimental Data in Trajectory Deviation Analysis

**DOI:** 10.3390/biomimetics10030138

**Published:** 2025-02-25

**Authors:** Jean Mendes Nascimento, Camila Taira, Eric Cito Becman, Arturo Forner-Cordero

**Affiliations:** Biomechatronics Laboratory, Escola Politécnica, University of Sao Paulo, São Carlos 13566-590, SP, Brazilaforner@usp.br (A.F.-C.)

**Keywords:** nonlinear control, neuromusculoskeletal model, star drawing, simulated precision task

## Abstract

Control remains a challenge in precision applications in robotics, particularly when combined with execution in small time intervals. This study employed a two-degree-of-freedom (2-DoF) planar robotic arm driven by a detailed human musculoskeletal model for actuation, incorporating nonlinear control techniques to execute a precision task through simulation. Then, we compared these simulations with real experimental data from healthy subjects performing the same task. Our results show that the Feedback Linearization Control (FLC) applied performed satisfactorily within the task execution constraints compared to a robust nonlinear control technique, i.e., Sliding Mode Control (SMC). On the other hand, differences can be observed between the behavior of the simulated model and the real experimental data, where discrepancies in terms of errors were found. The model errors increased with the amplitude and remained unchanged with any increase in the task execution frequency. However, in human trials, the errors increased both with the amplitude and, notably, with a drastic rise in frequency.

## 1. Introduction

Robotics have been used to assist humans in a huge variety of fields and tasks, such as industrial and medical applications [1], as they can reduce the muscular effort when performing a task [2]. Human–robot interfaces are becoming essential in advanced manufacturing, surgical robotics, and rehabilitation devices. Along with artificial intelligence, mechatronics, and technological advancements, robots provide flexibility for processes; however, they are still limited in terms of production and demand variation, fine handling, and manipulation. This limitation impairs their ability to operate effectively alongside humans in these contexts. Hence, addressing this challenge is crucial for improving the efficiency, safety, and overall performance of robotic systems across a wide range of applications [3,4,5,6].

Industry and factory workers are supposed to keep good pace and performance at work while handling heavy tools in improper positions, during a wide range of activities such as drilling, countersinking, electrical work, welding, grinding, picking, pruning, painting, inspection and overhead assembly [2,7], exposing them to many injuries risks. These activities are all examples of tasks that require some level of precision, coordination, and gross and fine motor skill frequently present in assembly lines.

A growing number of job positions are starting to be learned and performed by robots and cobots. Even though automation and mechanization are targeted in manufacturing assembly lines to optimize productivity along with robotics, human work is still needed and cannot be replaced or eliminated easily [6,8]. They may contribute with force input and speed, but, on the other hand, they are not yet able to perform fine handling and manipulation, and do not allow for the same flexibility and dexterity of human beings [3,4].

In this context, bioinspired solutions [9,10] have been implemented to improve the efficiency of these robotic systems in terms of reaction, adaptability, flexibility, robustness, and, above all, a stable and suitably compliant mechanical form regarding the complexity of such control. One key step toward overcoming this issue is the development of control models that accurately represent human motion control during these precision tasks [3].

As in several biological systems, the human motor system has excellent performance in terms of adaptability and robustness, and muscles show very low energy consumption [5]. In complex multi-joint movements, the precise coordination of multiple muscles is required to ensure smooth and controlled motion [11,12]. Thus, for robotics to be truly effective, assistive devices and their interfaces must be comfortable and natural to use. In that respect, a model of human motion control can guide the design of robotic systems that not only enhance human precision, but also avoid imposing unnatural movement or constraints. This could enable the robot to move in synchrony synergy with the user [1].

A musculo-skeletal model is a mathematical representation of the human body, including bones and muscles. It can be manipulated to estimate kinematics (e.g., joint trajectories) or dynamics (e.g., joint or muscle forces) under different conditions [13]. When robots work closely with humans in real time, their movements must be responsive and adaptive to ensure safe and effective interaction, accurately representing biomechanical and neuromuscular control [14]. By providing a better understanding of typical movement patterns, these models could generate predictive control signals that reduce delays in human–machine interactions [6]. Furthermore, models allow for the prediction of the behavior and analysis of the relative effects of each variable, distinguishing between expected and unexpected changes in motion, allowing robots to intervene when undesirable changes occur, potentially preventing errors or compensating for user fatigue [15].

Since several activities of daily living comprise multi-joint movements, such coordination between muscles is crucial for us to be functional and properly interact with the surrounding environment. The studies on biomechanics properties of the neuromuscular system in motor control are usually carried out applying mechanical perturbations to the limb during natural movements and observing the corrective responses [16]. Accurate human motion models could expedite the design and prototyping of biomimetic robotic systems by simulating expected human movement in various scenarios without requiring and relying on time-consuming experimental data. This could facilitate the development process and allow for more rapid testing and system refinement [17]. Some models of human biological systems that are frequently used in the study of human motor behavior are based on muscular physiology [18] and others are based on the neuromusculoskeletal mechanisms, which include the Golgi tendon organ (GTO) and muscle spindles [19].

However, current research often focuses on relatively simple tasks to test these models, such as single-joint or low-precision movements, which limits the applicability of these models in designing robotic systems intended for close human collaboration [20,21]. Thus, there is a need for models that capture and reproduce the control mechanisms of human motion during complex precision tasks.

Upon the establishment of the neuromusculoskeletal model [19] to be applied, two nonlinear control techniques were selected for implementation. From the framework of classical nonlinear control theories [22], a robust control strategy, specifically Sliding Mode Control (SMC), and a non-robust control method, namely Feedback Linearization Control (FLC), were prioritized for evaluation and subsequent performance analysis.

The three nonlinear control techniques most frequently applied in the literature are FLC, SMC, and MRAC (Model Reference Adaptive Controller). In this work, FLC was chosen since the model is fully known and it demonstrates compatible for this type of application, apart from its easiness of implementation. In addition, SMC was also implemented due to the fact that it is a robust control system; therefore, it can deal with unpredictable scenarios, such as when the mass parameters or any other nonlinearities present in the human body are not well known, thus it is not needed to be adaptive (MRAC). SMC can be compared with FLC (a technique of a different nature) to provide evidence of the best performance of the chosen high precision task.

In this work, we aimed to address this need by investigating the effectiveness of neuromusculoskeletal control models in capturing human arm motion during a multi-joint, high-precision task. Specifically, we focused on the task of drawing a star, which has been previously used in the literature as a representative precision activity for evaluating fine motor skills and control accuracy. Given the nonlinear nature of joint motions, we first explored two nonlinear control techniques—FLC and SMC—to determine which provides superior trajectory tracking for the star-drawing task. Subsequently, we compared these simulation results from both control models with experimental data from healthy subjects performing the same task [23].

We compared the tracking position error and transient time of two controllers, FLC and SMC. While both controllers had good tracking position performance, the FLC showed better transient performance. This model, along with the controller can be employed in the design of industrial and medical manipulators, such as artificial upper limbs, to predict the performance of an individual and it also brings efficiency to studies sparing time-consuming experiments in data collection. Such optimization may have a huge impact on the widespread acceptance of exoskeletons and other human-assistive devices by improving human–machine interface and interaction and by enhancing productivity and performance in industrial tasks. Finally, this work also contributes to a variety of future multidisciplinary studies involving biomechatronics, neuroscience, artificial intelligence, computer science, ergonomics, and rehabilitation altogether engaged in a long-lasting effort into human-inspired robotics [9].

## 2. Materials and Methods

### 2.1. Neuromusculoskeletal Model

To simulate human motion during precision tasks, we used the FLC and SMC control techniques to drive a neuromusculoskeletal model of the human arm. This neuromusculoskeletal model served as a biomechanical and physiological framework that the control models manipulated to replicate human joint motion.

Our neuromusculoskeletal model was based on [19], designed to investigate the roles of spinal proprioceptive feedback in movement control. This model was selected for its comprehensive representation of muscle activation and control mechanisms, providing a detailed parameter set that enhances the fidelity of our simulations.

The model encompasses muscle activation and control processes, including the elbow joint and a pair of antagonist muscles. It incorporates velocity and length feedback from muscle spindles, spinal stretch reflexes, reciprocal inhibition, and recurrent inhibition via Renshaw cells. Descending neural commands modulate the background activation of α-motoneuron pools in conjunction with these reflex activities, while static γ commands control spindle contraction [19].

**Figure 1:**
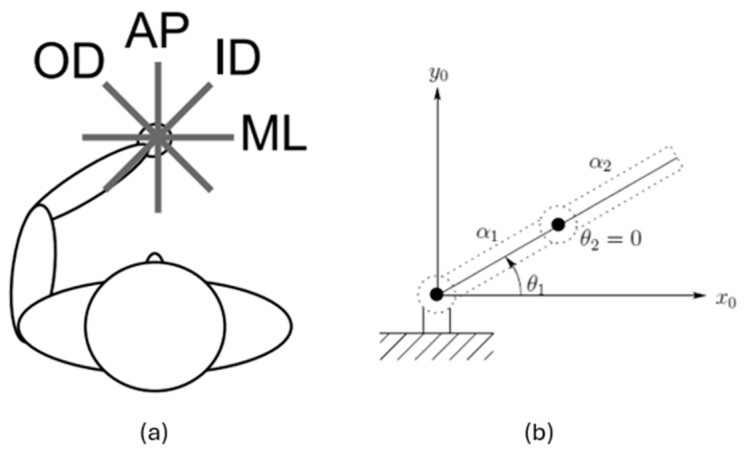
(**a**) Human arm as a planar two degrees-of-freedom to execute the task. Adapted from [23], and (**b**) two degrees-of-freedom mechanical robot model structure in comparison. Adapted from [24].

To depict human arm movements involving both the shoulder and elbow joints, we adapted the original model which works with only a single joint to a two-joint configuration, as seen in Figure 1, utilizing a two-degree-of-freedom (2-DoF) planar robotic arm representation, replicating the same joint activation in our model for both joints.

### 2.2. Control Techniques

We implemented two nonlinear control strategies to assess their effectiveness in trajectory tracking for the star-drawing task: Feedback Linearization Control (FLC) and Sliding Mode Control (SMC). These well-established control methods were selected based on their theoretical foundations in classical nonlinear control and their distinct operational characteristics.

Feedback Linearization Control was chosen because it effectively linearizes nonlinear system dynamics when the system model is fully known, which was the case in our simulations, and due to its relative simplicity and ease of implementation.

Sliding Mode Control was selected for its robustness to system uncertainties. This property is particularly valuable when dealing with the inherent nonlinearity and parameter variations in human biomechanics. SMC does not require adaptive mechanisms to handle such uncertainties, making it an appropriate choice for scenarios where system parameters may not be precisely known.

### 2.3. Simulation Procedure

Simulations were used to control the 2-DoF planar robotic arm (described by the Neuromusculoskeletal Model section) during a star-drawing task. In this task, explained in detail in section II-D, the robotic arm’s endpoint traces the lines of a star-shaped figure (see Figure 3d). The goal is to guide the arm along the lines of this star, reaching specific Cartesian coordinates that define its shape.

To achieve this, inverse kinematics is used to transform the Cartesian coordinates of the star into corresponding joint angles for the robotic arm. The inverse kinematics block receives a neuro-activation function over time, as modeled by [19]. Based on the planned trajectory path, it calculates the desired positions for each joint, $\theta_{1}$ and $\theta_{2}$, over time, following the methodology presented in [24]. These positions are then provided as set-points for the control system. Then, the selected control algorithms (FLC and SMC) are applied to ensure the arm accurately follows the desired joint trajectories.

Upon completing the simulated task, forward kinematics is used to convert the joint angles $\theta_{1}$ and $\theta_{2}$ back to Cartesian coordinates ($x_{1}$, $y_{1}$ and $x_{2},\;y_{2})$. This step is based on a relatively simple algebraic conversion due to the geometry of the problem [24] and is useful for visualization of the drawn star and facilitates a direct comparison between the intended trajectory and the one executed by the robotic arm under each control strategy.

To perform the star, the arm is initially fully extended and starts the required trajectory from position (2, 0), with the base positioned at point (0, 0), as seen in Figure 3d. The center of the star is located at (0.8, 0.8), and each straight-line segment forming the star measures exactly 70 mm. For this initial proof of concept, the dimensions of the arm segments, the size of the star, and the starting point were proportionally scaled but chosen arbitrarily.

### 2.4. Task Description

To assess joint control in multi-joint, high-precision tasks, we analyzed the movement involved in repeatedly drawing a star in the horizontal plane. In this task, the goal is to trace the lines of a given target star as accurately as possible while following specified amplitudes and frequencies of motion. This requires precise and coordinated control over multiple joints, making it appropriate for our study. While the human arm has more than two degrees of freedom, the primary movements in this task occur within the horizontal plane and involve only the shoulder and elbow joints. Consequently, this motion can be simplified and modelled as a 2-DoF system, focusing on these two joints, as in the neuromusculoskeletal model used in this article.

We analyzed both simulated data and real experimental data. These experimental data were sourced from the dataset used in [23], and our simulations were designed to replicate the experimental protocol of this dataset.

In the experimental setup [23], six subjects performed a series of movements to sketch a star-like figure composed of four intersecting lines with different orientations, forming an eight-pointed star in the horizontal plane, as can be seen in Figure 3d. Participants were seated in a stabilized position allowing only shoulder and elbow movements. They were instructed to trace the lines of the star with the tip of a low-friction stylus on a desk positioned in front of them.

For each of the four orientations—anteroposterior (AP), outer-diagonal (OD), mediolateral (ML), and inner-diagonal (ID) (Figure 1)—participants traced the corresponding line five times. Each repetition consisted of forward and backward movements that began and ended at the center of the star. After completing five repetitions for a given orientation, participants proceeded to the next orientation in a clockwise order for right-handed individuals or counterclockwise for left-handed individuals. Upon completing all four orientations, participants repeated the process once more in the same order. Completing each orientation twice constituted a single trial. Trials were conducted continuously without interruptions between orientations or repetitions. Participants were instructed to trace the lines as precisely as possible, synchronizing the beginning of each repetition with a metronome beat.

The dataset included multiple trials executed by volunteers using two different motion amplitudes (8 cm and 16 cm, measured between two opposite extremities of the star) and two repetition frequencies (1.33 Hz and 2.00 Hz, guided by a metronome). Each complete trial lasted around 31 or 21 s, depending on the frequency used. For each amplitude-frequency combination, four separate trials were conducted, resulting in a total of 16 trials per individual (2 amplitudes × 2 frequencies × 4 trials).

These four conditions—combinations of amplitude and frequency—allow for an analysis of the effects of movement amplitude and frequency on motor control and precision. Accordingly, we conducted our simulations under these same four conditions to enable a direct comparison between the experimental data and the simulated results.

### 2.5. Joint Control

For a joint *j* and time *t*, its dynamic properties are modelled as follows:

(1) $$I_{j}\frac{d^{2}\theta }{dt^{2}}+B_{j}\frac{d\theta }{dt}=T_{j}$$

Being,

(2) $$B_{j}=0.03\sigma$$

where *Ij* is the moment of inertia, *Bj* is the coefficient of viscosity, *Tj* is the resultant torque and *θ* is the joint angle. To calculate the coefficient of viscosity, the joint stiffness (σ) is necessary and is calculated as presented in [19], as well as all the dynamic parameters used.

The first control technique applied on the joint system was Feedback Linearization Control. In this case, being *u* the control input of the system, *u* is given as follow [22]:

(3) $$u=\frac{1}{b}\;[\;v-f]$$

where *v* can be any linear control technique, *b* and *f* are auxiliary equations to linearize the system. In this work, *v* is a Proportional-Derivative (PD) controller applied combined with pole allocation to obtain the gain values K_1_ and K_2_, as in:

(4) $$v={\ddot{\theta }}_{d}-K_{1}\dot{\tilde{\theta }}-K_{2}\tilde{\theta }$$

(5) $$\tilde{\theta }=\theta -\theta_{d}$$

The adequate values to K_1_ and K_2_ were identified experimentally according to the desired task.

In another way (1) can be rewritten as

(6) $$\ddot{\theta }=I_{j}^{-1}\;(T_{j}-B_{j}\dot{\theta }\;)$$

Being identified *f* and *b* as auxiliary equations:

(7) $$f\left(\dot{\theta }\right)=I_{j}^{-1}\;(-B_{j}\dot{\theta })$$

(8) $$b=I_{j}^{-1}$$

Thus, the input torque was defined as

(9) $$u=T_{j}=I_{j}\;[v+B_{j}\dot{\theta }\left(I_{j}^{-1}\right)]$$

And, the resulting linearized system is

(10) $$\ddot{\theta }=v$$

The Sliding Mode Control technique was also applied to the nonlinear joint system. Being the so-called robust control, the sliding mode is recommended when the model parameters are uncertain. Let (5) be the tracking error in the variable θ.

For the robust sliding mode, a time-varying sliding surface *s(θ,t)* = 0 is defined as the expression [22]

(11) $$s\left(\theta ,t\right)={\left(\frac{d}{dt}+\lambda \right)}^{n-1}\;\tilde{\theta }=0$$

where λ is a parameter to be tuned strictly positive constant and *n* refers to the system order. In the case *n* = 2. Hence, we have

(12) $$s=\dot{\tilde{\theta }}+\lambda \tilde{\theta }$$

(13) $$s\left(\theta ,t\right)=\left(\frac{d}{dt}+\lambda \right)\tilde{\theta }=\left(\dot{\theta }-{\dot{\theta }}_{d}\right)+\lambda \;\left(\theta -\theta_{d}\right)$$

Being $\dot{s}$ calculated as follows:

(14) $$\dot{s}=\left(\ddot{\theta }-{\ddot{\theta }}_{d}\right)+\lambda \;\left(\dot{\theta }-{\dot{\theta }}_{d}\right)=f\left(\theta ,\dot{\theta }\right)+b\left(\theta \right)U-{\ddot{\theta }}_{d}+\lambda (\dot{\theta }-{\dot{\theta }}_{d})$$

As $\dot{s}$ = 0 and f has the same parameters used in the Feedback Linearization Control, then

(15) $$\hat{u}=\frac{1}{\hat{b}}\left[-\hat{f}+{\ddot{\theta }}_{d}-\lambda \left(\dot{\theta }-{\dot{\theta }}_{d}\right)\right]$$

Hence, $\hat{u}$ depends on $\hat{f}$ and $\hat{b}$,which are estimates of (5) and (6). Thus, $\hat{u}$ is the best estimate of the equivalent control and the final controller is given by

(16) $$u=\hat{u}-K\;sgn(s)$$

where sgn is the sign function which adds robustness against disturbances, being $sgn\left(s\right)=+1$ if s>0 or $sgn\left(s\right)=-1$ if s<0 and *K* is a positive constant that must be chosen to be large enough, defined as:

(17) K≥F−η

In this sense, η is a tuned value influenced directly by $t_{r}$, that is how long the trajectory takes to reach the sliding surface s and *F* is the maximum error given by the modeling, considered uncertainty parameters, defined as

(18) $$\eta =\frac{s(0)}{t_{r}}$$

(19) $$F=f-\hat{f}$$

## 3. Results

The initial simulations sought to assess the performance of the two proposed controllers. The first aspect analyzed for the given precision task was the controller’s ability to reach some arbitrary prescribed angles. Another fundamental factor for the task was the transient time, that is, the time it takes for the controller to completely reach a desired point. Since the frequencies for performing the task developed by [23] were varied, a high transient time means more time for the system to reach the desired points and, consequently, for changes in direction [22].

**Figure 2:**
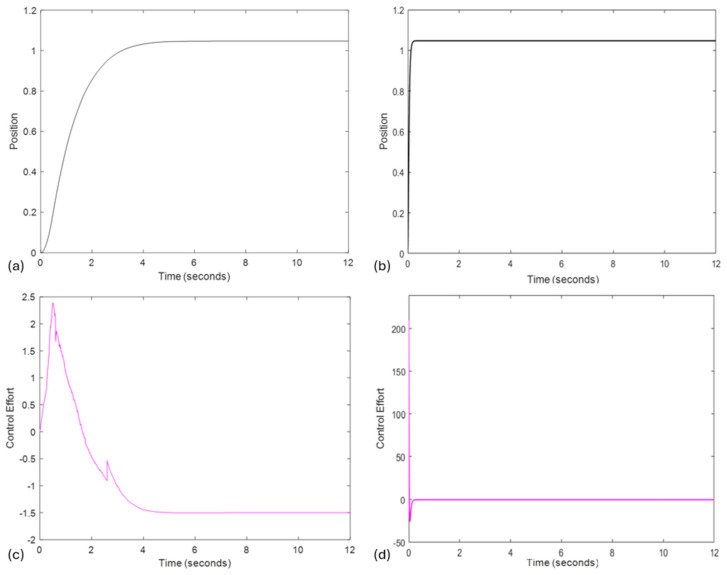
Comparison between Sliding Mode Control (SMC) and Feedback Linearization Control (FLC) controllers. (**a**) Performance of the SMC controller, (**b**) performance of the FLC controller, (**c**) SMC input control effort, and (**d**) FLC input control effort.

Figure 2 presents a comparison between both controllers reaching a set-point of π/3 rad/s, which was arbitrarily defined. Specifically, Figure 2a depicts the performance of the Sliding Mode Control (SMC) controller, while Figure 2b illustrates the performance of the Feedback Linearization Control (FLC) controller. Additionally, the behavior of the controllers is shown in Figure 2c for SMC and Figure 2d for FLC.

The following tests were carried out in order to provide the star drawing task. The system controlled by SMC was also tested. However, in the initial test, it did not perform as expected for the task. As shown in Figure 2a, the system approached the desired position closely after t = 5 s but only fully reached the target position after t = 8.65 s. Due to this limitation, further analyses were discontinued.

**Figure 3:**
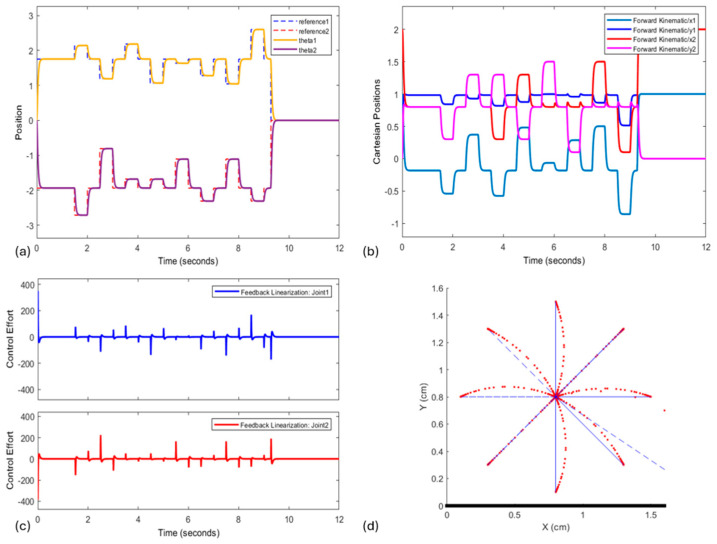
Feedback Linearization Control behavior. (**a**) Tracking position of star coordinates; (**b**) Cartesian coordinates from forward kinematic; (**c**) controller behavior; and (**d**) star drawing result from Feedback Linearization Control.

Figure 3 shows the FLC initial results carrying out the task according to the desired inputs. With the positions provided by inverse kinematics, Figure 3a shows the tracking response for each joint angle required to draw the star, as presented in Figure 3d. Figure 3b represents the Cartesian positions obtained through forward kinematics for each joint, which are necessary and allow to plot the star’s trajectory, and Figure 3c illustrates the controller’s behavior for each joint throughout the system’s operation. Finally, Figure 3d shows the completed task and final star representation.

**Figure 4:**
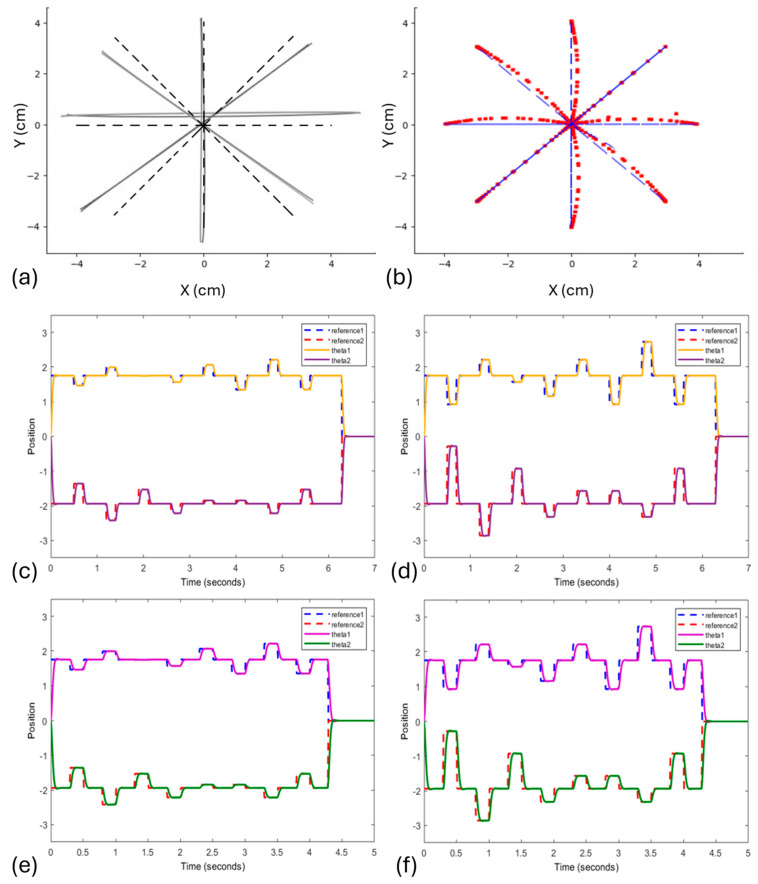
(**a**) Mean of star drawing task with an amplitude of 8 cm and frequency of 1.33 Hz from [23]; (**b**) task from the FLC controller with the same configuration; (**c**–**f**) tracking position with a frequency and amplitude of 1.33 Hz/8 cm, 1.33 Hz/16 cm, 2.00 Hz/8 cm and 2.00 Hz/16 cm, respectively.

In order to validate the bioinspired task with the controller, a comparison was carried out between the results obtained by the FLC technique and the real data results obtained by the experiment in [23]. For comparison purposes, the trajectories made by the subjects who participated in the experiment were combined into an average, where Figure 4a shows the star drawing task with an amplitude of 8 cm and frequency of 1.33 Hz.

In comparison, Figure 4b presents the task from the model controlled with the same configuration of Figure 4a. Complementary to this, Figure 4c–f shows the tracking position of the task with a frequency and amplitude of 1.33 Hz/8 cm, 1.33 Hz/16 cm, 2.00 Hz/8 cm and 2.00 Hz/16 cm, respectively. 

**Figure 5:**
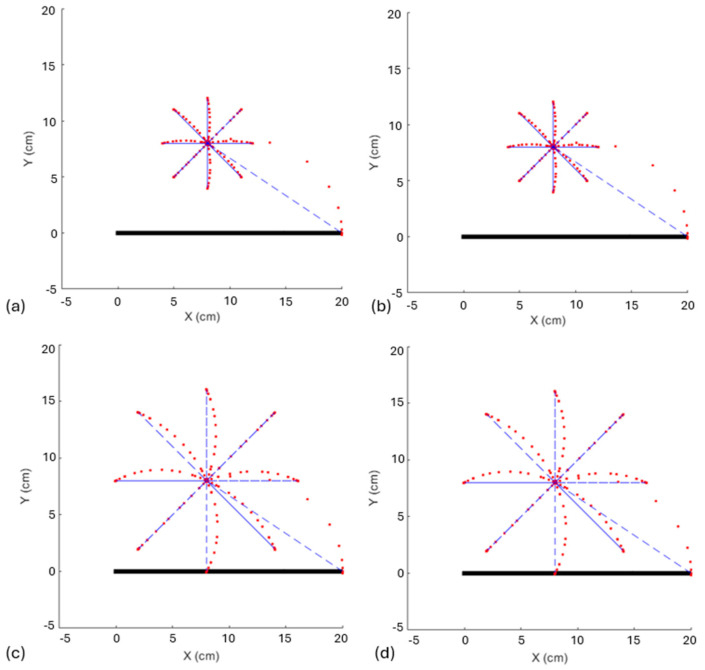
Tasks with same amplitudes and frequencies employed in the experiment conducted by [23]. (**a**) Star with an amplitude of 8 cm and a frequency of 1.33 Hz; (**b**) star drawn with the same amplitude of 8 cm but at a frequency of 2.00 Hz; (**c**,**d**) amplitude of 16 cm at the frequencies of 1.33 Hz and 2.00 Hz, respectively.

Figure 5 presents the results of the task, utilizing the same amplitudes and frequencies employed in the experiment conducted by [19]. Figure 5a illustrates the resulting star with an amplitude of 8 cm and a frequency of 1.33 Hz, while Figure 5b shows a star drawn with the same amplitude of 8 cm but at a frequency of 2.00 Hz. Additionally, Figure 5c,d displays stars with an amplitude of 16 cm at the frequencies of 1.33 Hz and 2.00 Hz, respectively.

**Figure 6:**
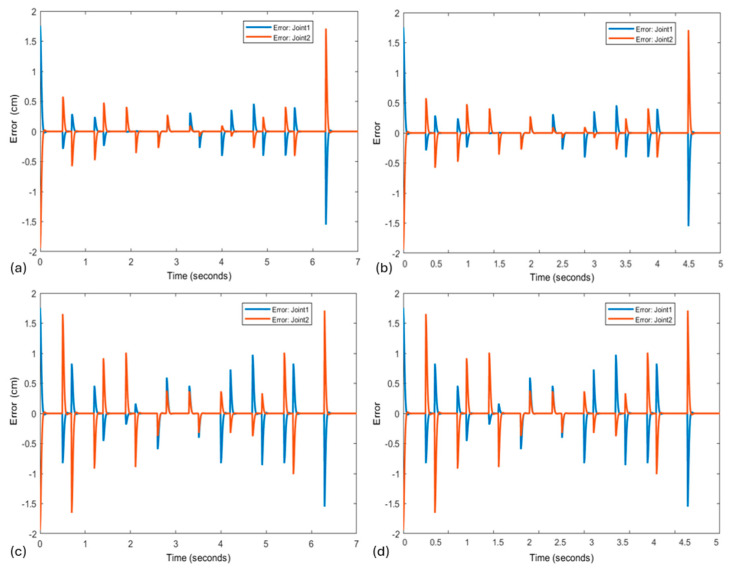
Trajectory errors for each star and their respective amplitude and frequency: (**a**) star with an amplitude of 8 cm and a frequency of 1.33 Hz; (**b**) error for the same amplitude but at a frequency of 2.00 Hz; (**c**,**d**) errors for increased amplitude of 16 cm, corresponding to the frequencies of 1.33 Hz and 2.00 Hz, respectively.

Consequently, Figure 6 presents the corresponding trajectory errors for each tested star, based on its amplitude and frequency. Figure 6a shows the trajectory error for the star generated with an amplitude of 8 cm and a frequency of 1.33 Hz, while Figure 6b presents the error for the same amplitude but at a frequency of 2.00 Hz. In contrast, Figure 6c and Figure 6d illustrate the trajectory errors for stars with an increased amplitude of 16 cm, corresponding to the frequencies of 1.33 Hz and 2.00 Hz, respectively.

## 4. Discussion

In this work, a simulated control of the human arm was proposed. Firstly, the neuromusculoskeletal model presented in [19] was simulated. This model allowed the muscle system to act as the human body actuator in a 2-DoF planar robotic manipulator configuration. In the end of the proposed task, the controlled system should draw a star and the results were compared with the study carried out by [23] with humans.

The model classified by [19,25] as spinal neuromusculoskeletal is composed of a complete model of muscle activation considering the neural signals, also known as the muscle spindle. The spindle is a sensory organ found in most vertebrates and it plays a crucial role both in kinesthesia and in reflexive adjustments to perturbations. Moreover, it is the main source of proprioceptive feedback for spinal sensorimotor regulation and servocontrol [26].

Patients without proprioceptive stimulation from their limbs have difficulties performing multi-joint movements. For example, in hand gestures that require sharp direction reversals of the hand path, these patients present large deviations on the hand path, due to failure to coordinate the timing of the distinct reversals in the shoulder joints and elbow [27]. Therefore, the importance of the spindle in muscle control, thus movement control, and path corrections is evident, which makes it essential to be considered in the muscle model, as previously shown in other works [19,25], and since the task applied in this work is considered a precision task where the feedback and path corrections are crucial.

The Feedback Linearization Control (FLC) performed very well but was unable to provide tracking performance that reached all angles along the task trajectory (Figure 3a,d). The controller can accurately reach the desired points, but an error can always be observed when there is a change in direction along the trajectory. Human motion in the experimental data [23] also presents deviation during star-drawing trajectory execution. Due to the kinematics of the robotic arm applied within the model, such trajectory deviation was not a result of mechanical singularities, it must be on the account of our model being human-based. Singularity, in this case (2-DoF planar robot), would be only if the robotic arm was fully flexed or extended, which was not the case. And, since the system was modeled considering fully known parameters, i.e., dynamic uncertainties were not taken into consideration, the application of disturbances would lead to a decrease in the performance of the system controlled by the FLC.

The Sliding Mode Control (SMC) presented satisfactory tracking behavior, showing capacity to achieve desired angles and expected stability, but it presented a very long transient state, which was due to the input parameters. When they were changed, in order to decrease the transient time, the consequence was an overshooting, which is even worse regarding task accuracy, as the deviation was larger and more frequent. However, unlike the FLC system, when disturbances were introduced into the system in this case, the controller’s performance remained unaffected, as it was designed considering dynamic uncertainties.

The artificial controllers were compared according to two criteria, tracking position error and transient time. In the tracking position, both controllers showed good results. But in the transient state that was adopted as another criterion, a better performance of Feedback Linearization Control was observed.

The results presented by the FLC combined with the experiment set up from [23] showed that increasing the task amplitude also increased the error (Figure 5a and Figure 6a versus Figure 5c and Figure 6c, Figure 5b and Figure 6b versus Figure 5d and Figure 6d), but when the frequency was increased, the error did not change (Figure 5a and Figure 6a versus Figure 5b and Figure 6b, Figure 5c and Figure 6c versus Figure 5d and Figure 6d). This behavior differed from the same test with humans, where when the amplitude increased, the error increased as well, but when the frequency increased, the error increased even more (Figure 6 versus Figure 2 in [23]). This result was expected, since humans are unconsciously subject to several biological factors that influence feedback information necessary to correct these errors.

Once we know how and which control performs better in a model, comparing it with human real data, we can start applying this control to design bioinspired industrial manipulators, artificial upper limbs, to predict the expected performance of an individual without physiological interferences and without the need for time-consuming experimental data.

Several studies have investigated such collaborative human–robot interactions [28,29,30,31]. There are other types of precision tasks that simulate industrial work and rehabilitation exercises, for example, timing coincident [32,33] and drilling [8,34]. The star drawing task assesses precision on both the vertical and horizontal plane, timing coincident however, just on the horizontal plane. An advantage is that besides vertical and horizontal traces, the start drawing also includes diagonal traces in four directions, northeast to southwest, and northwest to southeast; that is why this task is also called “compass drawing” since it covers the total of four range traces (north to south, east to west and both diagonals).

Between the applied controls, FLC parameters are changeable, which allows personalization by replacing them in the code. In this way, we can predict the best performance of the arm from different subjects and compare it with real data. Afterward, it would be possible to study and investigate trajectory deviations caused by other robotic devices such as industrial and medical exoskeletons or obtain a reference to be achieved by the patient in rehabilitation protocols.

While our work opens a path to simulate how an individual would perform this precision task in optimal circumstances without any interference, physiological or due to the exoskeleton use, future work shall include testing exoskeletons while performing the same task, aiming to analyze where and what disturbances are caused by the use of these devices. Furthermore, it would be possible also to analyze other human condition interferences such as sleep deprivation, line assembly at a fast pace, fatigue effects, besides workspace and its environment, different exoskeleton models, and inappropriate posture.

Our work comes to analyze how well a model can perform a precision task through the simulation and control technique. If robots could have similar dexterity of humans via control, their spread can go further than augmenting force and mainly speed in industrial tasks. This ability to perform precision work in even shorter time would be another advantage, especially in terms of productivity. Additionally, we are to benefit from this introduced skill to improve our interaction with them, optimizing processes.

Our contribution would be toward knowing the best human performance for the star drawing task. In this way, we can predict the best arm performance of different subjects without having to perform tests and experiments on this task. Moreover, we will be able to recognize trajectory deviations caused by robotic assistive devices such as exoskeletons and provide a reference for exercise goals for patients in rehabilitation protocols. Not only can this control be employed in the design of industrial and medical manipulators, such as artificial upper limbs, to predict the expected performance of an individual free from physiological or external interferences, but it also brings efficiency to studies, sparing time-consuming experiments in data collection.

Such optimization may have a huge impact on the widespread acceptance of exoskeletons and other human-assistive devices by improving the human–machine interface and interaction, and by enhancing productivity and performance in industrial tasks. 

What the industry and rehabilitation have in common is the fact that in both areas, exoskeletons, as a personal assistance device, can be used in a wide range of contexts, industry types and medical protocols, several tasks and exercises, apart from benefiting a variety of working population and patients [35]. Control, fluid mobility, and consequent better functionalities that are still an issue for their development can be delivered through improved and more robust control strategies [36]. This is exactly what we are looking for in the present work.

## 5. Conclusions

In this study, first, we present an upper limb control analysis, considering only the elbow and shoulder joints on the horizontal plane, followed by requirements for this control. After the bioinspired mechanical model to be controlled is explained, we tested two nonlinear control techniques in the model, and we applied only the one that performed better (FLC) to control the proposed arm in the star drawing task. We compared them with real data, and an unexpected behavior was seen in terms of errors. The model errors increased with the amplitude and remained unchanged with any increase in the task execution frequency. However, in human trials, the errors increased both with the amplitude and, notably, with a drastic rise in frequency. Therefore, it is possible to establish a foundation for the development of future robotic devices, whether for industrial applications or rehabilitation, which, despite being based on biomimetic concepts, demonstrate superior performance in precision tasks under constrained conditions tested with human subjects.

## Data Availability

The data presented in this study are available on request from the corresponding author due to the need to maintain data privacy while continuing work with future tests and experiments.

## References

[1] Zhao Y., Wu H., Zhang M., Mao J., Todoh M. (2023). Design methodology of portable upper limb exoskeletons for people with strokes. Front. Neurosci..

[2] Pinho J.P., Forner-Cordero A. (2022). Shoulder muscle activity and perceived comfort of industry workers using a commercial upper limb exoskeleton for simulated tasks. Appl. Ergon..

[3] Spada S., Ghibaudo L., Gilotta S., Gastaldi L., Cavatorta M.P. (2017). Investigation into the Applicability of a Passive Upper-limb Exoskeleton in Automotive Industry. Procedia Manuf..

[4] Pesenti M., Antonietti A., Gandolla M., Pedrocchi A. (2021). Towards a functional performance validation standard for industrial low-back exoskeletons: State of the art review. Sensors.

[5] Fox S., Aranko O., Heilala J., Vahala P. (2020). Exoskeletons: Comprehensive, comparative and critical analyses of their potential to improve manufacturing performance. J. Manuf. Technol. Manag..

[6] Mukherjee D., Gupta K., Chang L.H., Najjaran H. (2022). A Survey of Robot Learning Strategies for Human-Robot Collaboration in Industrial Settings. Robot. Comput. Integr. Manuf..

[7] Weston E.B., Alizadeh M., Knapik G.G., Wang X., Marras W.S. (2018). Biomechanical evaluation of exoskeleton use on loading of the lumbar spine. Appl. Ergon..

[8] Kim S., Nussbaum M.A., Mokhlespour Esfahani M.I., Alemi M.M., Alabdulkarim S., Rashedi E. (2018). Assessing the influence of a passive, upper extremity exoskeletal vest for tasks requiring arm elevation: Part I–“Expected” effects on discomfort, shoulder muscle activity, and work task performance. Appl. Ergon..

[9] Qiao H., Zhong S., Chen Z., Wang H. (2022). Improving performance of robots using human-inspired approaches: A survey. Sci. China Inf. Sci..

[10] Panizzolo F.A., Galiana I., Asbeck A.T., Siviy C., Schmidt K., Holt K.G., Walsh C.J. (2016). A biologically-inspired multi-joint soft exosuit that can reduce the energy cost of loaded walking. J. Neuroeng. Rehabil..

[11] MacDougall W. (2014). Industrie 4.0: Smart Manufacturing for the Future.

[12] Baldassarre A., Lulli L.G., Cavallo F., Fiorini L., Mariniello A., Mucci N., Arcangeli G. (2022). Industrial exoskeletons from bench to field: Human-machine interface and user experience in occupational settings and tasks. Front. Public Health.

[13] Lewis C.L., Sahrmann S.A., Moran D.W. (2009). Effect of position and alteration in synergist muscle force contribution on hip forces when performing hip strengthening exercises. Clin. Biomech..

[14] Su H., Qi W., Chen J., Yang C., Sandoval J., Laribi M.A. (2023). Recent advancements in multimodal human–robot interaction. Front. Neurorobot..

[15] Scibilia A., Pedrocchi N., Fortuna L. (2022). Human Control Model Estimation in Physical Human–Machine Interaction: A Survey. Sensors.

[16] Lenzi T., Vitiello N., McIntyre J., Roccella S., Carrozza M.C. (2011). A robotic model to investigate human motor control. Biol. Cybern..

[17] Li Q., Zhang Z., You Y., Mu Y., Feng C. (2020). Data Driven Models for Human Motion Prediction in Human-Robot Collaboration. IEEE Access.

[18] Hill A.V. (1970). First and Last Experiments in Muscle Mechanics.

[19] Lan N., Li Y., Sun Y., Yang F.S. (2005). Reflex regulation of antagonist muscles for control of joint equilibrium position. IEEE Trans. Neural Syst. Rehabil. Eng..

[20] Chen J., Zhong S., Kang E., Qiao H. (2019). Realizing human-like manipulation with a musculoskeletal system and biologically inspired control scheme. Neurocomputing.

[21] Wu Y., Yuan J., Qiao H. (2024). Equilibrium-Point Control and Robustness Analysis of Bioinspired Musculoskeletal Robotic System. IEEE/ASME Trans. Mechatron..

[22] Slotine J.-J.E., Li W. (1991). Applied Nonlinear Control.

[23] Levin O., Forner-Cordero A., Li Y., Ouamer M., Swinnen S.P. (2008). Evidence for adaptive shouder-elbow control in cyclical movements with amplitudes, frequencies, and orientations. J. Mot. Behav..

[24] Spong M.W., Hutchinson S., Vidyasagar M. (2006). Robot modeling and control. IEEE Control Syst..

[25] Yong Li N.L., Yang F. Feedback regulation of joint equilibfuum states with alpha-gamma Coactivation. Proceedings of the 19th Annual International Conference of the IEEE Engineering in Medicine and Biology Society. ‘Magnificent Milestones and Emerging Opportunities in Medical Engineering’ (Cat. No.97CH36136).

[26] Mileusnic M.P., Brown I.E., Lan N., Loeb G.E. (2006). Mathematical models of proprioceptors. I. Control and transduction in the muscle spindle. J. Neurophysiol..

[27] Sainburg R.L., Ghilardi M.F., Poizner H., Ghez C. (1995). Control of limb dynamics in normal subjects and patients without proprioception. J. Neurophysiol..

[28] Mendes Nascimento J., Dos Santos Brito C., Davi Castro Frazão E., Santos Mera G.A., Alves Aniceto da Silva G., Venceslau de Melo H.G. (2022). Robotic arm inertial control for recreational child physiotherapy application. Int. J. Adv. Med. Biotechnol. IJAMB.

[29] Papanagiotou D., Senteri G., Manitsaris S. (2021). Egocentric Gesture Recognition Using 3D Convolutional Neural Networks for the Spatiotemporal Adaptation of Collaborative Robots. Front. Neurorobot..

[30] Antonaci F.G., Olivetti E.C., Marcolin F., Castiblanco Jimenez I.A., Eynard B., Vezzetti E., Moos S. (2024). Workplace Well-Being in Industry 5.0: A Worker-Centered Systematic Review. Sensors.

[31] Choi S.H., Park K.B., Roh D.H., Lee J.Y., Mohammed M., Ghasemi Y., Jeong H. (2022). An integrated mixed reality system for safety-aware human-robot collaboration using deep learning and digital twin generation. Robot. Comput. Integr. Manuf..

[32] Forner-Cordero A., Quadrado V.H., Tsagbey S.A., Smits-Engelsman B.C.M. (2018). Improved learning a coincident timing task with a predictable resisting force. Motor Control.

[33] De Melo G.C., Martes Sternlicht V., Forner-Cordero A. EEG Analysis in Coincident Timing Task Towards Motor Rehabilitation. Proceedings of the Annual International Conference of the IEEE Engineering in Medicine and Biology Society, EMBC.

[34] Alabdulkarim S., Kim S., Nussbaum M.A. (2019). Effects of exoskeleton design and precision requirements on physical demands and quality in a simulated overhead drilling task. Appl. Ergon..

[35] Howard J., Murashov V.V., Lowe B.D., Lu M.L. (2020). Industrial exoskeletons: Need for intervention effectiveness research. Am. J. Ind. Med..

[36] Rupal B.S., Rafique S., Singla A., Singla E., Isaksson M., Virk G.S. (2017). Lower-limb exoskeletons: Research trends and regulatory guidelines in medical and non-medical applications. Int. J. Adv. Robot. Syst..

